# The No-Win Resuscitation: Ventricular Septal Rupture and Associated Acute Aortic Occlusion

**DOI:** 10.1155/2018/1568491

**Published:** 2018-11-19

**Authors:** Jan-Thorben Sieweke, Jens Vogel-Claussen, Andreas Martens, Jörn Tongers, Andreas Schäfer, Johann Bauersachs, L. Christian Napp

**Affiliations:** ^1^Cardiac Arrest Center, Department of Cardiology and Angiology, Hannover Medical School, Hannover, Germany; ^2^Institute for Diagnostic and Interventional Radiology, Hannover Medical School, Hannover, Germany; ^3^Department of Cardiothoracic, Transplantation and Vascular Surgery, Hannover Medical School, Hannover, Germany

## Abstract

A 66-year-old patient was admitted under continuous resuscitation for pulseless electrical activity. After return of spontaneous circulation ECG showed signs of acute inferior ST-elevation myocardial infarction, and echocardiography showed acute right ventricular failure with a dilated right ventricle. Carotid pulses were present in the absence of femoral pulses. Subsequent computed tomography demonstrated inferior myocardial infarction with ventricular septal rupture and thrombotic occlusion of the thoracic aorta, resulting in a heart-brain-circulation with loss of perfusion downstream of the aortic arch.* Teaching Points.* The present case prototypically demonstrates the fatal consequence of acute ventricular septal rupture and the eminent value of computed tomography and palpation of carotid in addition to femoral pulses in resuscitated patients. It is, to the best of our knowledge, the first description of an acute aortic occlusion in a patient with acute ventricular septal rupture.

## 1. Introduction

Prognosis of out-of-hospital cardiac arrest remains poor [1], despite loads of efforts in emergency and critical care medicine. Major goals to improve outcome are early return of spontaneous circulation (ROSC) with only minimal no-flow-time prior to and during cardiopulmonary resuscitation (CPR), and early detection of the cause of arrest, paralleled by optimal post-resuscitation care. Efficacy of chest compressions and ROSC are usually monitored by palpation of pulses, commonly femoral pulses, in addition to capnography. However, in selected cases palpation of femoral pulses only is not reliable, either with coexisting peripheral artery disease or with rare conditions as in our case. After ROSC a dedicated diagnostic and therapeutic program has to be started to improve outcomes. At this stage the diagnostic pathway should also include computed tomography (CT), as already practiced by an increasing number of large centers, for determining the cause of arrest and to uncover important comorbidities. In our case the CT scan inadvertently uncovered a fatal diagnosis.

## 2. Case Presentation

**Figure 1:**
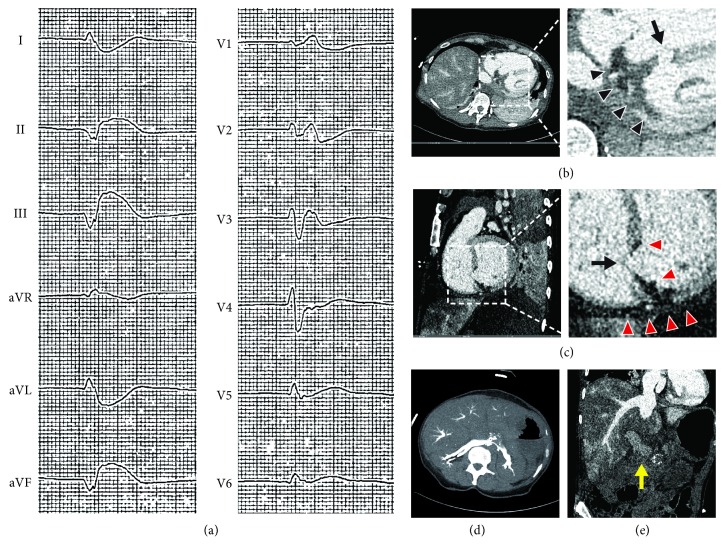
**Signs and consequences of ventricular septal rupture due to acute myocardial infarction.** (a) ECG. ST-segment elevation in leads II, III, aVF, and V6. (b) to (e): Computed tomography. (b) Transverse plane. Acute inferior myocardial infarction (black arrowheads) and ventricular septal rupture (black arrow), both associated with dilation of the right ventricle and retrograde flow of contrast agent into the liver veins due to acute right heart failure. (c) Sagittal plane. Ventricular septal rupture (black arrow) due to acute myocardial infarction with extensive microvascular obstruction (red arrowheads). (d) Transverse plane, arterial phase after intravenous injection of contrast agent. Contrast is seen in renal and hepatic veins as a sign of severe venous congestion. (e) Sagittal oblique plane, late phase after injection of contrast agent. Hepatic veins, hepatic parenchyma, and the portal vein (yellow arrow) are still opacified due to backward failure of the right heart.

**Figure 2:**
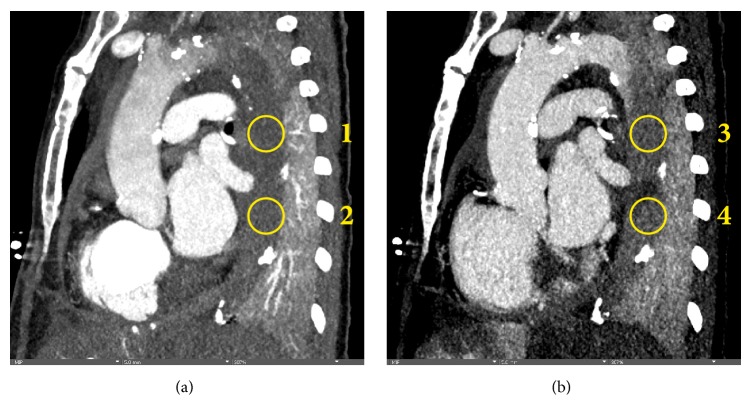
**Acute aortic occlusion. **Computed tomography. (a) Sagittal plane, arterial phase after intravenous injection of contrast agent. The descending aorta is not filled with contrast (circle 1: 70 HU, circle 2: 73 HU). Note the severe calcification of the aorta. (b) Sagittal plane, late phase after intravenous injection of contrast. While few contrast enters the proximal part of the thrombus (apposition zone, circle 3: 105 HU), it does not reach the already more solid parts of the thrombus (circle 4: 78 HU). HU: Hounsfield Units.

A 66-year-old female patient, who was hospitalized in a psychiatric clinic, suddenly developed dysarthria and anisocoria with subsequent loss of consciousness. Without palpable pulses immediate bystander CPR was performed and the patient was transferred to our hospital. On the way CPR of the ventilated patient was continued with a mechanical resuscitation device (LUCAS 2, Physio-Control™). After 45 minutes of CPR for pulseless electrical activity spontaneous circulation returned as assessed by a palpable carotid pulse, however femoral pulses were absent, precluding cannulation for mechanical circulatory support (MCS). ECG showed ST-segment elevation in leads II, III, aVF, and V6 (Figure 1). Fast-track echocardiography demonstrated severe dysfunction of the dilated right ventricle. Pericardial effusion and severe aortic regurgitation were absent. Medical history comprised an infrarenal aortic aneurysm. Acute aortic dissection Stanford Type A, De Bakey I with involvement of the right coronary artery was suspected. As percutaneous MCS was not possible without femoral pulses and since Type-A-dissection would have prompted emergency surgery, the heart team decided for immediate computed tomography (CT), which showed no signs of Type A aortic dissection, pulmonary embolism, intracerebral bleeding, or carotid stenosis, but severe ubiquitous aortic calcification. Main findings were as follows: a large ventricular septal rupture with classical radiological signs of acute myocardial infarction (Figure 1), signs of severe backward failure with contrast agent being observed in hepatic veins, renal veins (Figure 1), the portal vein and the inferior vena cava (Figure 1), and thrombotic occlusion of the descending aorta just distal to the left subclavian artery (Figure 2). The latter was probably facilitated by massive left-to-right shunt and associated severe forward failure. Shortly after the CT scan the patient had to be resuscitated again, but due to futility with subtotal body ischemia without any option for MCS or emergent surgery resuscitation was terminated.

## 3. Discussion

Acute ventricular septal rupture is one of the most dangerous complications of myocardial infarction [2, 3]. In patients with large septal defects and conservative management, mortality is unacceptably high with rates up to 100% [4]. Therefore, those patients are classic candidates for urgent surgery, and recently results of interventional closure are even more encouraging, with survival rates reaching over 50% [5]. In this context CT has emerged as the gold standard for assessment of anatomy before treatment [4].

Our patient had an acute ventricular septal rupture due to acute inferior myocardial infarction resulting in right ventricular failure and low cardiac output syndrome. In such cases MCS is an important option as a bridge to surgery or intervention [6, 7], which was not feasible due to proximal aortic occlusion. The massive left-to-right shunt maybe does not solely explain aortic occlusion. Additional plaque rupture of a diseased aorta or local dissection associated with CPR may have been the initial injury triggering local thrombosis. Alternatively, classic aortic dissection Stanford Type B, De Bakey III may have been present subsequently resulting in thrombosis. Furthermore, tissue embolization from septal rupture could have been the initial trigger for thrombus formation. However, CT findings were consistent with a very large thrombus without signs of aortic dissection or tissue embolism, thus suggesting that low cardiac output was the predominant determinant of the developing occlusion. Unfortunately, confirmation of those findings would have required autopsy, which was declined by the patient's family. In addition efficacy of mechanical CPR may have been limited in this particular patient due to the left-to-right shunt, which could have been a reason for low flow in the aorta triggering subsequent thrombosis.

Notwithstanding, the absence of femoral pulses in the presence of carotid pulses after CPR should prompt a CT scan, as in our case. Fast-track echocardiography in the emergency room after ROSC did not uncover the VSR in our patient, maybe due to the very basal position of the defect. Without aortic occlusion, the patient would have had a chance to survive - with the use of MCS, septal closure, coronary intervention, and appropriate post-cardiac arrest care. Irrespective of the outcome in this single case, CT would have been a prerequisite for treatment planning.

To the best of our knowledge, the present case is the first to illustrate an acute occlusion of the descending aorta in a patient after out-of-hospital cardiac arrest. It corroborates the extraordinary role of palpating all major pulses and echocardiography during cardiac arrest management, as well as the value of computed tomography after ROSC, either to search for therapeutic options or to detect fatal situations in order to terminate therapy.

## Data Availability

The data (ECG and CT images, and CT densitometry) used to support the findings of this study are included within the article. CT raw data of this study are restricted by institutional policies in order to protect patient privacy and therefore cannot be made publicly available.
